# A Case of Delayed Airway Stenosis Due to Retropharyngeal Hematoma Caused by Low Energy Trauma

**DOI:** 10.7759/cureus.26087

**Published:** 2022-06-19

**Authors:** Issei Tanaka, Takeshi Arizono, Akihiko Inokuchi, Ryuta Imamura, Teiyu Izumi

**Affiliations:** 1 Orthopaedics, Kyushu Central Hospital of the Mutual Aid Association of Public School Teachers, Fukuoka, JPN; 2 Orthopaedic Surgery, Kyushu Central Hospital of the Mutual Aid Association of Public School Teachers, Fukuoka, JPN

**Keywords:** hyperextension of the neck, delayed onset, airway obstruction., retropharyngeal hematoma., low-energy trauma

## Abstract

Airway narrowing due to trauma-induced retropharyngeal hematoma is rare. However, it is dangerous to overlook this lesion because it can lead to airway obstruction and even death. In this article, we report a case of a patient who developed pharyngeal pain and dysphagia two days after bruising on the forehead due to a fall and required intubation management.

A 52-year-old man fell while walking and bruised his forehead two days before visiting our hospital. He had a sore throat and dysphagia two days after the injury and came to our hospital three days after the injury. The swelling was observed in the anterior neck, and stenotic sounds were heard in the upper airway. Cervical CT and MRI of the cervical spine showed extensive hyperabsorption areas in the ventral side of the cervical spine that appeared to be hematomas. No fracture of the cervical spine was observed. The patient has been placed on emergency tracheal intubation due to concerns about airway stenosis caused by the hematoma. Although pneumonia was observed during treatment, it resolved with antimicrobial therapy, and the hematoma tended to shrink, so the patient was extubated on the 15th day of admission. However, the patient was intubated again on the 17th day of hospitalization due to poor oxygenation. A tracheostomy was performed on the 26th day of hospitalization due to suspected narrowing of the upper airway caused by hematoma or sputum. On day 59 of hospitalization, the cannula was removed, and the patient was discharged home on the 68th day after hospitalization.

Low-energy trauma tends to be underrecognized as producing anterior cervical hematomas that can lead to fatal airway narrowing. Care should be taken because fatal anterior cervical hematomas are not often part of the differential diagnosis due to their often delayed onset. More caution is needed if an underlying disease may cause coagulation abnormalities.

## Introduction

Trauma-induced retropharyngeal hematoma is relatively rare [1]. However, its anatomical location can cause airway obstruction, and even minor trauma can lead to death, deep neck infection, and mediastinitis, so urgent treatment is necessary. It is not easy to diagnose if the onset is delayed after the injury. In this article, we report a case of a patient who developed pharyngeal pain and dysphagia two days after a fall and bruising on the forehead and had upper airway stenosis due to retropharyngeal hematoma.

## Case presentation

**Figure 1:**
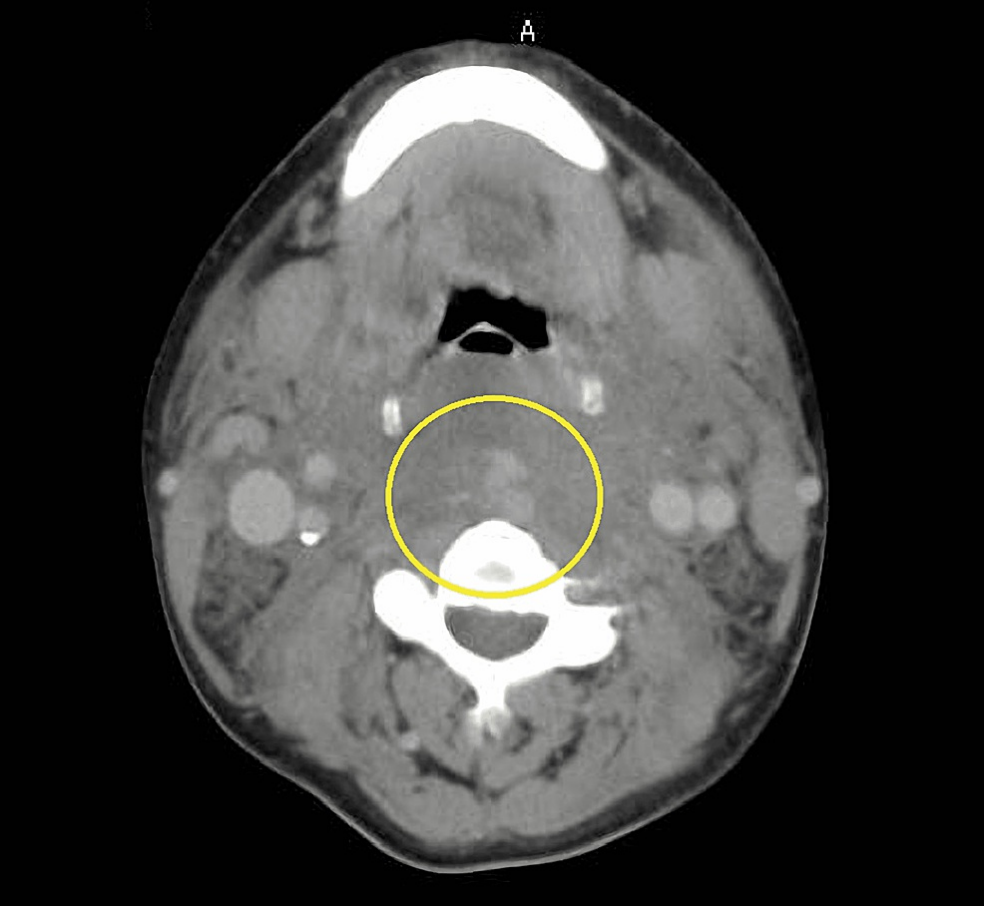
Cervical contrast CT at initial examination (axial) Swelling of the retropharynx is noted.

**Figure 2:**
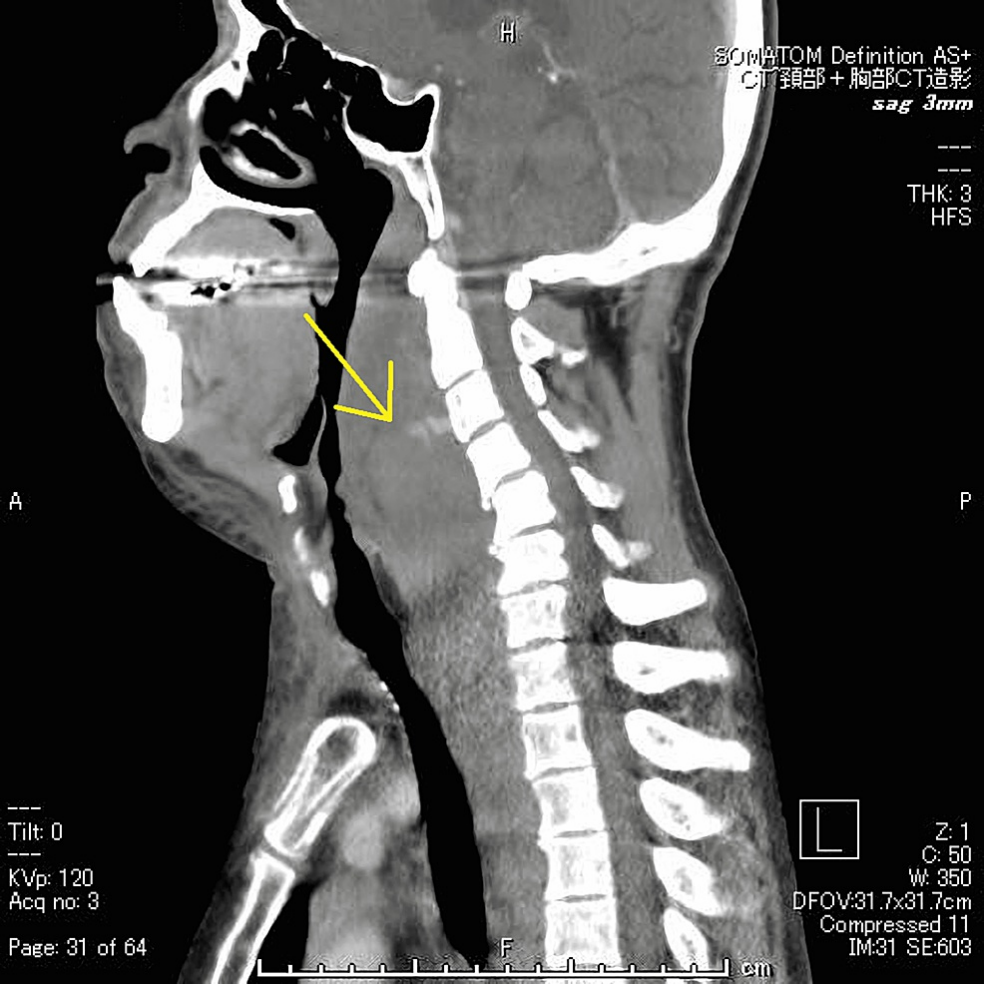
Cervical contrast CT at initial examination (sagittal) The arrow points to the high-density area, which was considered a hematoma.

A 52-year-old man who fell while walking and bruised his forehead came to our emergency room on the third day after his injury. When he bruised his forehead, his neck was hyperextended. The patient's consciousness was lucid. He complained of sore throat, dysphagia, and anterior neck swelling. Physical examination revealed anterior cervical swelling and fever. In addition, upper airway stenosis sounds were heard. There was no paralysis or sensory disturbance in the extremities. A lung murmur wasn't heard bilaterally. He could speak but could not swallow spit, which was painful. His temperature was 36.7°C, blood pressure 150/103 mmHg, pulse 138/min (regulated), respiratory rate 12/min, and oxygen saturation 98% (room air). Initial laboratory results showed a white blood cell count of 8,100 /µL, Hb of 12.5 g/dL, platelet count of 85,000 /µL, albumin of 2.4 g/dL, total bilirubin of 2.3 mg/dL, C-reactive protein (CRP) of 0.12 mg/dl, and prothrombin (PT) activity of 52.6 %. Cervicothoracic contrast-enhanced CT showed extensive, highly resorbed structures on the ventral side of the cervical spine that appeared to be hematomas (Fig 1, 2).

**Figure 3:**
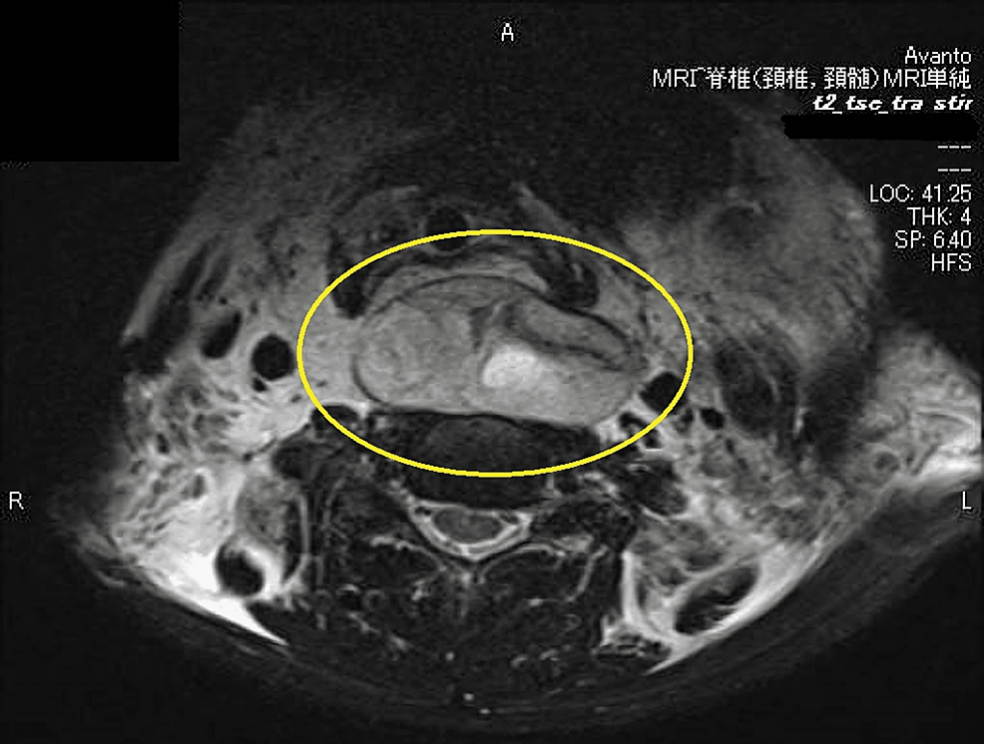
Cervical spine MRI at initial examination (axial) MRI also shows swelling of the retropharynx.

**Figure 4:**
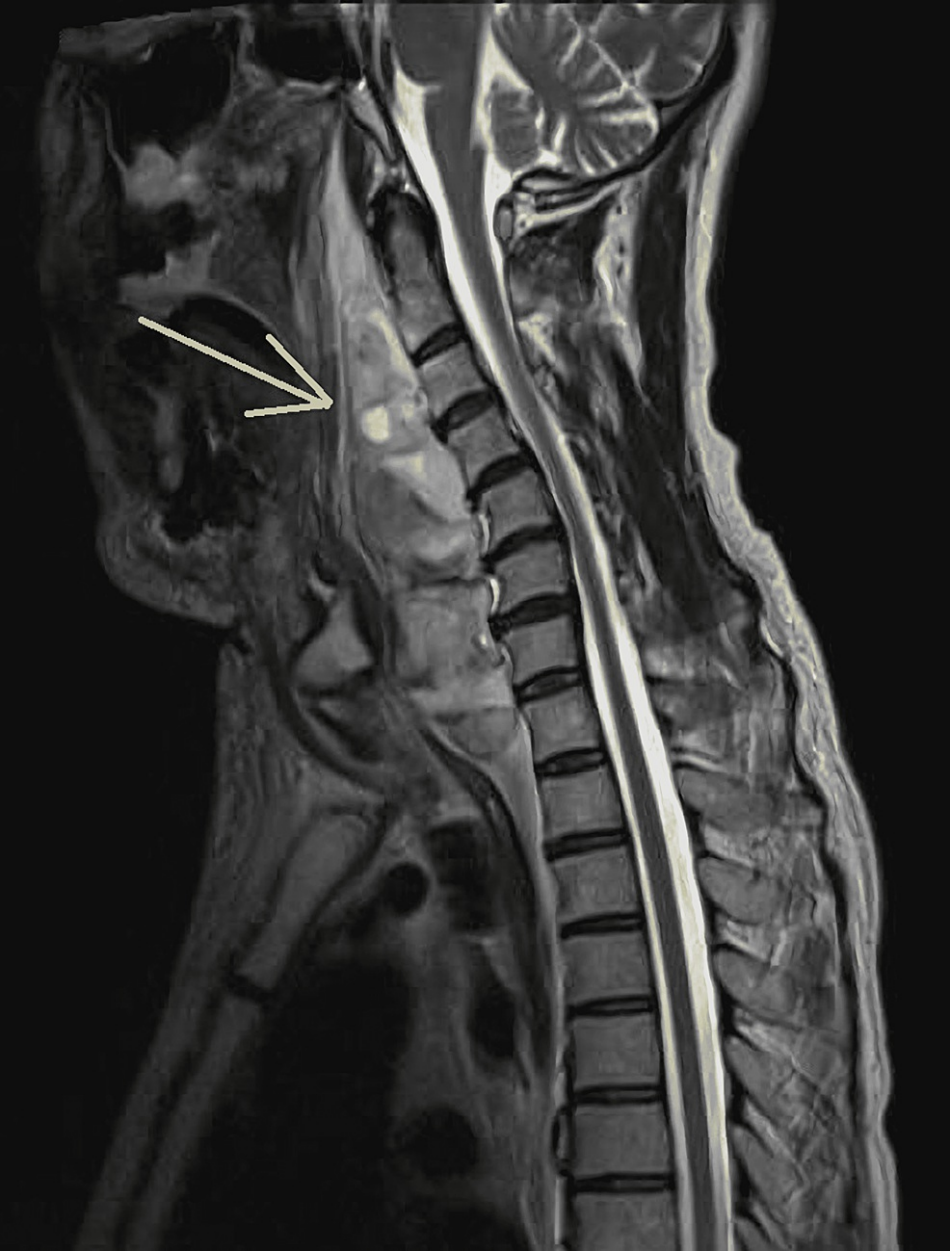
Cervical spine MRI at initial examination (sagittal) No obvious cervical fracture is seen. The arrow indicates a high-density area, which extends over the anterior cervical spine and is thought to be a hematoma.

A structure that appeared to be a pseudoaneurysm was observed on the ventral side of the 2nd-3rd cervical vertebra. It was determined that upper airway stenosis due to the hematoma might develop in the future and that intubation and respiratory management were indicated to secure the airway. The larynx was expanded using a McGrath laryngoscope, and intubation was performed using a bronchoscope. He had a history of alcoholic cirrhosis, hypertension, reflux esophagitis, and a postoperative left inguinal hernia incarceration and was not on anticoagulants or antiplatelet agents. The patient had a platelet count of 85,000, PT activity of 52.6%, and markedly decreased coagulation capacity. MRI scan of the cervical spine was performed (Fig 3, 4).

**Figure 5:**
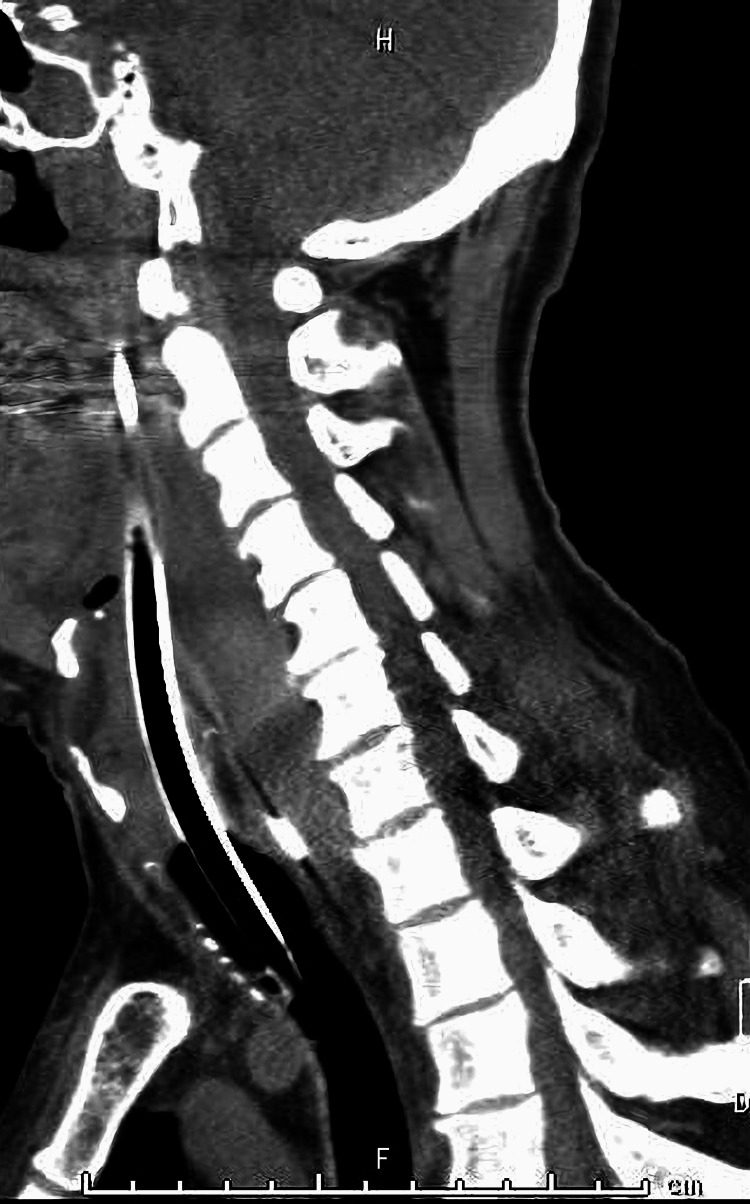
Contrast-enhanced CT scan on the 15th day of hospitalization Compared to the initial examination, the hematoma has shrunk.

There was no obvious fracture of the cervical spine. Soft structures extended over the anterior cervical spine, and the pharynx and larynx were compressed. Internally, T1WI showed equal to mildly high-intensity lesions, and T2WI showed heterogeneous high-intensity lesions, and fluid level formation was seen in some areas. STIR high-intensity lesion extended into the mediastinum and cervical interstitium. It was thought to be a cervical perivertical hematoma. On day five of hospitalization, the patient developed a fever in the 38°C range, judged as pneumonia, and was treated with antimicrobial agents from the 7th to 15th day of hospitalization. Contrast-enhanced CT scan on the 15th day of hospitalization showed a reduction in the size of the hematoma (Fig 5).

After performing a cuff leak test, the patient was extubated with no dyspnea, no airway narrowing sounds, and no change in oxygenation (SpO_2_: 95% oxygen at 5L/min). However, on the morning of day 17 of hospitalization, SpO_2_ dropped to 30%, and the patient was urgently intubated and managed again. A tracheostomy was performed on the 26th day of hospitalization because we suspected that the hematoma may not have shrunk sufficiently and that there was a phlegm blockage. We anticipated the need for long-term airway management. On day 46 of hospitalization, the patient was switched to a speech cannula, and on day 51, swallowing function training was started. The cannula was removed on day 59 of hospitalization, and the patient was discharged home on day 68.

## Discussion

Causes of retropharyngeal hematoma include neck trauma, neck surgery, deep neck infection, anticoagulant therapy, cannulation, foreign body ingestion, retropharyngeal infection, carotid artery aneurysm, and even cough and vomiting [2-4]. Among these, retropharyngeal hematoma secondary to blunt trauma is very rare, with only about 63 cases reported as far as we referred [3-5]. This may be because they are more common in elderly patients at higher risk of falling due to decreased physical function, decreased support of the cervical spine, and in many cases taking anticoagulants and antiplatelet medications [6].

The most common symptom at presentation is dyspnea, with other symptoms including neck pain, hoarseness, and dysphagia. Cervical swelling is often present. It has been reported that airway obstruction symptoms appeared within 1 hour of injury in less than 40% of cases [7]. The possibility of a delayed appearance of a retropharyngeal hematoma should be considered.

Based on history, symptoms, local findings such as neck swelling, and imaging studies, diagnosis is relatively easy if the symptoms occur after injury. However, with delayed symptoms, as in our case, it is sometimes challenging to suggest the hematoma due to injury. Contrast-enhanced CT shows a mixture of high-, iso-, and low-absorption areas in the acute phase of the disease, but the low-absorption areas gradually increase. An accurate and prompt diagnosis is vital to obtaining a good prognosis.

Because of the narrowing of the upper airway, the initial treatment of retropharyngeal hematoma is to secure the airway [8]. Subsequent treatment is conservative therapy with careful observation, sometimes requiring emergency surgery. If the stenosis is severe, tracheostomy or bronchoscopic intubation is recommended because of the risk of hematoma enlargement or rupture due to intubation maneuvers. Conservative treatment was chosen again in this case because it is considered reasonable to wait for spontaneous resorption unless the hematoma is markedly enlarged or does not disappear for an extended period, as some reports indicate no difference in treatment time between conservative treatment and hematoma removal. In many cases, hematomas have been reported to regress spontaneously in 2 to 4 weeks [8].

The patient had a relatively low-energy head contusion that resulted in neck hyperextension. The patient developed pharyngeal pain and dysphagia on the second day of injury, and a delayed retropharyngeal hematoma was noted. Thus, the progression of symptoms was slow. On day 15 of hospitalization, the hematoma was found to have shrunk, and the patient was extubated but required reintubation two days later. It was considered possible that the hematoma had shrunk sufficiently, had grown again, or that the patient could not expectorate sputum properly, resulting in sputum collection. The cannula was removed on the 59th day of hospitalization, a more extended period to secure the airway than in other reported cases. The patient had a history of alcoholic cirrhosis and decreased coagulation capacity, which may have contributed to the fact that it took longer than usual for the hematoma to regress. In some cases, cooperation with physicians is considered necessary. This case showed that it is vital to recognize that retropharyngeal hematoma may appear later, even with low-energy cervical hyperextension.

## Conclusions

We have experienced a case in which a relatively low-energy head contusion resulted in neck hyperextension, and a delayed pharyngeal hematoma caused airway stenosis. Low-energy trauma can cause fatal symptoms such as delayed hematoma and dyspnea. Therefore, it is crucial always to be suspicious of such cases, especially when an underlying disease causes abnormalities in the coagulation system.
